# Association of Tumor-Infiltrating Lymphocytes and Inflammation Status with Survival Outcome in Patients with High-Grade Serous Ovarian Carcinoma

**DOI:** 10.3390/cancers17142269

**Published:** 2025-07-08

**Authors:** Simona Miceska, Cvetka Grašič Kuhar, Snježana Frković Grazio, Erik Škof, Praveen Krishnamoorthy, Dineo Khabele, Veronika Kloboves Prevodnik

**Affiliations:** 1 Department of Cytopathology, Institute of Oncology, Zaloska cesta 2, 1000 Ljubljana, Slovenia; smiceska@onko-i.si; 2 Faculty of Medicine, University of Ljubljana, Vrazov trg 2, 1000 Ljubljana, Slovenia; cgrasic@onko-i.si (C.G.K.); eskof@onko-i.si (E.Š.); 3 Department of Gynecology and Obstetrics, Washington University School of Medicine in St. Louis, 4911 Barnes Jewish Hospital Plaza, St. Louis, MO 63110, USA; khabeled@wustl.edu; 4 Department of Medical Oncology, Institute of Oncology, Zaloska cesta 2, 1000 Ljubljana, Slovenia; 5 Department of Pathology, Gynecology Clinic, University Clinical Centre Ljubljana, Šlajmerjeva ulica 3, 1000 Ljubljana, Slovenia; snjezana.frkovicgrazio@kclj.si; 6 Washington University Center for Cellular Imaging WUCCI, Washington University School of Medicine in St. Louis, St. Louis, MO 63110, USA; praveen.krishnamoorthy@wustl.edu; 7 Faculty of Medicine, University of Maribor, Taborska Ulica 8, 2000 Maribor, Slovenia

**Keywords:** immunohistochemistry, inflammation status, high-grade serous ovarian carcinoma, digital-image analysis, manual scoring, tumor-infiltrating lymphocytes, prognostic biomarkers

## Abstract

**Background/Objectives**: Tumor-infiltrating lymphocytes (TILs) and inflammation status are emerging prognostic markers in various cancers, but their significance in high-grade serous ovarian carcinoma (HGSC) remains unclear. Our objective was to evaluate different TIL subtypes and inflammation status in relation to progression-free survival (PFS) in primary HGSC. **Methods**: CD3^+^/CD4^+^/CD8^+^/PD-1+ stromal TILs (sTILs) and intraepithelial TILs (iTILs) were evaluated by manual assessment and digital image analysis (DIA), following TIL Working Group recommendations. Inflammation status was evaluated through the following scores: systemic immune-inflammation index (SII), pan-immune-inflammation value (PIV), CA125, and lactate dehydrogenase (LDH). **Results**: CD8^+^ TILs were the most prevalent subtype in both iTILs and sTILs. However, sTILs were significantly more abundant than iTILs (*p* < 0.001) among all subsets, except for PD-1+ cells. DIA results of TIL assessments were in agreement with manual assessments. High stromal CD3^+^ and CD8^+^ TILs, PIV, CA125, and LDH, were associated with improved PFS. Potential independent prognostic factors for PFS in manual assessment were PIV (HR = 0.32, CI 95% = 0.12–0.82) and CD8^+^ sTILs (HR = 0.30, CI 95% = 0.12–0.79), whereas in DIA assessment they were CD3^+^ sTILs (HR = 0.31, CI 95% = 0.15–0.67), PIV (HR = 0.35, 95% CI 0.13–0.96), and residual disease (HR = 0.21 95% CI 0.08–0.53). **Conclusions**: CD3^+^/CD8^+^ sTILs and PIV are promising prognostic indicators in HGSC; however, further research is needed to confirm their clinical utility.

## Simple Summary

In this study, we investigated the association between tumor-infiltrating lymphocytes (TILs), inflammation status, and progression-free survival (PFS) in patients with primary high-grade serous ovarian carcinoma (HGSC). We assessed the percentages of different intraepithelial and stromal TIL subtypes using both manual and digital methods, following established recommendations for TIL assessment. In addition, we evaluated inflammation status through several immune scores, including the pan-immune-inflammation value (PIV). Our results suggest that stromal CD3^+^ and CD8^+^ TILs, as well as PIV, may serve as potential prognostic indicators in HGSC, as they remained potential independent markers in multivariate analysis.

## 1. Introduction

High-grade serous ovarian carcinoma (HGSC) is the most prevalent and aggressive form among the five epithelial ovarian carcinoma (EOC) subtypes, with a 5-year survival rate of less than 40% [1]. Due to its subtle symptoms, it is often diagnosed at an advanced stage (FIGO III or IV) when the disease has already spread to the peritoneum outside of the pelvis, lymph nodes, or distant organs [2]. Little progress has been made in improving the survival rates of HGSC in recent decades, highlighting the urgent need for novel prognostic biomarkers to more accurately assess the risk of disease progression and guide treatment strategies.

Tumor-infiltrating lymphocytes (TILs) have attracted considerable attention in recent years due to their favorable impact on patient survival in HGSC. In 2003, Zhang et al. were the first to report that the presence of CD3^+^ TILs correlated with delayed recurrence and improved overall survival in EOC patients, pointing out the role of endogenous anti-tumor immunity [3]. However, several years later, Sato et al. found that CD8^+^ TILs, rather than CD3^+^ TILs, were associated with improved survival, with no significant correlation observed for CD3^+^ TILs [4]. This discrepancy was partly explained by subsequent studies showing that while CD8^+^ TILs are indeed associated with better survival, CD4^+^ TILs are often associated with poorer survival, contributing to the different results regarding CD3^+^ TILs [5]. Moreover, the expression of programmed cell death 1 (PD-1) protein on TILs is also associated with unfavorable prognosis [6].

Although the number of studies on this topic is increasing, the data are still contradictory, mainly due to the different assessment methods and different EOC subtypes included. TILs have been assessed mostly on hematoxylin and eosin (H&E) and immunohistochemistry (IHC) slides in whole-tissue sections or microarrays, with some studies focusing on hotspots while others used randomly selected areas [7,8,9,10]. In addition, different scoring criteria are used to express the results, such as absolute counts or semi-quantitative assessments with different cut-off values [7,8,9,10]. It is important to note that the localization of TILs appears to be prognostically important, whether they are located in the tumor bed or on the periphery [11]. These differences appear not only in EOCs but also in other malignancies. Therefore, in 2017, the International Immuno-Oncology Biomarkers Working Group (TIL Working Group, TIL-WG) focused on developing recommendations to ensure the consistency of TIL assessment in standard histopathologic practice, research, and clinical trials, primarily in breast cancer, but also applicable in various other cancers. The TIL-WG defined “intra-epithelial” (iTILs) as TILs present in the tumor and “stromal” (sTILs) as TILs present within 1 mm below the epithelial layer, and suggested reporting iTIL and sTIL results separately, as percentages of surface area occupied by TILs while observing the slides in 3–10 IHC fields at 200× or 400× high-power fields (HPFs) and avoiding hotspots [12,13]. While these suggestions have provided valuable insights for ongoing studies, some uncertainties persist; nonetheless, the interest in this topic continues to grow. Notably, digital image analysis tools are emerging and could play a key role in the future of standardizing TIL assessments by providing more consistent and objective results [14,15,16].

Additionally, systemic inflammation, which promotes an immunosuppressive microenvironment, may reduce the infiltration of TILs in the tumor and tumor stroma, particularly CD8^+^ cells, and favor tumor progression. For instance, inflammatory biomarkers such as the neutrophil count, lymphocyte count, platelet count, neutrophil/lymphocyte ratio (NLR), platelet/lymphocyte ratio (PLR), and systemic immune-inflammation index (SII) have been reported to be related to patient survival and have prognostic value in various malignancies [17]. SII, in particular, has gained attention as it reflects both immune activation and suppression, making it a more comprehensive biomarker of systemic inflammation [18]. Recently, a new prognostic biomarker, the pan-immune-inflammation value (PIV), which includes neutrophil, platelet, monocyte, and lymphocyte counts, has been integrated, as it was found to be superior to other immune-inflammatory biomarkers [19]. Several other immune-related biomarkers, such as Cancer Antigen 125 (CA 125), lactate dehydrogenase (LDH), and C-reactive protein (CRP), which are easily accessible for evaluation, may reflect the immunosuppressive environment and potentially lead to T-cell exhaustion [20,21]. However, the prognostic effect of inflammation status on survival in ovarian cancer patients remains poorly understood, especially in HGSC, as only a limited number of studies are available and some results remain contradictory.

Our study had three primary objectives: (1) to accurately determine the percentages of CD3^+^, CD4^+^, and CD8^+^ iTILs and sTILs, and PD-1 expression in patients with HGSC at the time of diagnosis following the recommendations of the TIL-WG, further supported by digital image analysis; (2) to investigate the status of the inflammation, including SII, PIV, CA 125, LDH, and CRP; and (3) to investigate the association between TILs and inflammation status, as well as their association with patient survival.

## 2. Materials and Methods

### 2.1. Patients

Patients suspected of primary HGSC between January 2019 and May 2021 at the Institute of Oncology Ljubljana (IOL) and/or University Medical Centre Ljubljana (UMCL) were eligible for inclusion. Only those with histologically confirmed primary HGSC were included in the study. The extended inclusion criteria were as follows: age >18 years, WHO performance status from 0 and 1, histologically confirmed primary HGSC, presence of malignant ascites, indication for first-line systemic treatment with platinum agents, and having available follow-up data. All patients received standard treatment (chemotherapy and surgery) in accordance with the European Society for Medical Oncology (ESMO) guidelines [22]. Written informed consent for participation in the study was obtained from every patient.

### 2.2. Study Design

For the purpose of this study, an experienced gynecopathologist (S.F.G.) reviewed all H&E slides for each patient to select one representative slide that contained tumor tissue. From this selected slide, the corresponding formalin-fixed, paraffin-embedded (FFPE) tumor tissue block was retrieved from the Pathology Archives at IOL and UMCL. Five sections, each 4 μm thick, were then cut from this block for immunohistochemical (IHC) staining of CD3, CD4, CD8, PD-1, and a negative control to evaluate CD3^+^, CD4^+^, CD8^+^ and PD-1+ iTILs and sTILs by manual scoring under a light microscope, following the recommendations of the TIL-WG [12,13]. As standardized criteria for assessing TILs in HGSC are still lacking, a digital image analysis was performed as an additional method to support the manual scoring. Therefore, after the manual scoring was completed, the IHC slides were scanned at the Pathology Department of UMCL, generating NDPI format scans. These scans were used for the digital assessment of the TILs with the artificial intelligence (AI)-driven precision pathology software VisioPharm (VisioPharm, Hørsholm, Denmark), specifically trained for our analysis at Washington University Center for Cellular Imaging (WUCCI). The results of the two assessment methods were compared. Later, data on inflammation status at diagnosis, including platelet, neutrophil, lymphocyte, and monocyte counts to calculate SII and PIV, as well as data on CA125, LDH, and CRP levels, were retrieved from the patient’s medical record at IOL. TILs and inflammation status were stratified into high and low categories using receiver operating characteristic (ROC) curve analysis and then correlated with progression-free survival (PFS), alongside the analysis of FIGO stage and residual disease after surgery, with both univariate and multivariate analyses performed to assess these associations.

### 2.3. Immunohistochemistry

IHC staining was performed using the following antibodies: CD3 (clone LN10, 1:500, Leica Biosystems, Wetzlar, Germany), CD4 (clone SP35, 1:10, Cell Marque, Rocklin, CA, USA), CD8 (clone 144B, 1:100, Agilent, Santa Clara, CA, USA), and PD-1 (Nat105, 1:200, Cell Marque, Rocklin, CA, USA). The presence of the antigens was detected with an OptiView Ventana detection kit and BenchMark Ultra autostainer (Ventana, Roche Diagnostics, Oro Valley, Arizona). Enzymatic detection of the antibodies was accomplished with a secondary goat anti-mouse and anti-rabbit IgG conjugated to haptenylated secondary antibody (HQ), followed by an anti-HQ conjugated to horseradish peroxidase multimer (HRP). Chromogen was deposited by a reaction with hydrogen peroxide in the presence of diaminobenzidine (DAB) and copper sulfate, producing brown precipitate. The secondary antibodies, HRP multimer, and all chromogen reagents were applied at the instrument’s default times. Negative control slides omitting the primary antibody were included in all batches. Sections from tonsillar and placenta tissue served as positive control for CD3, CD4, and CD8.

### 2.4. Manual Scoring of iTILs and sTILs

The evaluation of CD3, CD4, CD8, and PD-1 IHC reaction was adapted from the standardized method described in Saldago et al. [12]. iTIL and sTIL compartments were scored separately, and the result is given as a percentage of intratumoral/stromal surface area covered by TILs. Values were estimated as the average percentage of area covered by CD3-, CD4-, CD8-, or PD-1-positive cells relative to total cell area, based on three randomly selected high-power fields (HPFs) at 400× magnification.

The manual evaluation was performed independently by two investigators (S.F.G. and S.M.) using a multi-view microscope. The more experienced investigator (S.F.G.) quickly looked at each whole slide under the microscope and selected the 3 most representative high-power fields (HPFs). This approach ensured that the same HPFs were analyzed for both evaluators, although the scoring was conducted independently. In case of discrepant results, a consensus was reached. Because other WBC populations (mainly macrophages) occasionally stain with CD4 antibody, CD4-positive mononuclear immune cells with an apparent morphological appearance different from T cells were excluded from the count.

### 2.5. Digital Image Analysis

The same slides used for the manual evaluation were scanned to NDPI files with the NanoZoomer Hamamatsu C13220 digital slide scanner (Hamamatsu Photonics, Hamamatsu, Shizuoka Prefecture, Japan) at the Institute of Pathology, UMCL, and were later analyzed with the VisioPharm 2021.02 platform (VisioPharm, Hørsholm, Denmark) at Washington University Center for Cellular Imaging, St. Louis, MO, USA.

**Figure 1:**
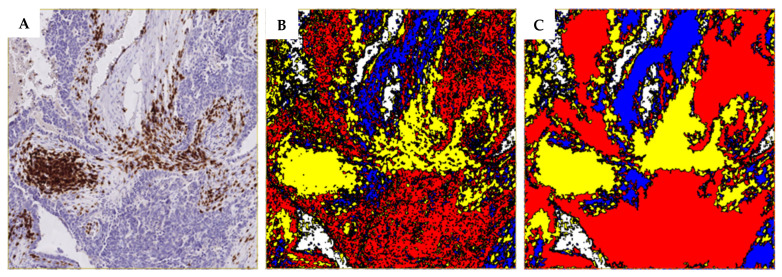
Visual explanation of the APP training process for digital image analysis. (**A**) The first image represents an immunohistochemically (IHC) stained tumor tissue-block section, where areas of interest are labeled with different colors: yellow indicating DAB-positive staining (for CD3, CD8 or PD-1), red representing tumor areas, and blue representing the stromal areas. (**B**) The second image represents the training of the APP to distinguish between these areas with high precision by repeatedly re-analyzing the differences between the colored areas. (**C**) shows the processing after the APP was properly trained, where the three-color IHC section was modified and converted into numerical data for further analysis. Images are given at 50× magnification.

Using an application (APP) module of the VisioPharm software, a smart learning algorithm was developed for digital image analysis. Due to the large image sizes and time-consuming process, a whole region of the tissue section visible on the screen at 5× magnification was randomly selected for further analysis. Regions of interest were defined within the APP, including tumor, stroma, DAB-positive cells, and background. The APP was then trained to accurately recognize and distinguish different cells in each of these regions. If there were any discrepancies between the regions in the samples, the APP was retrained. A preview tool was used to confirm the reliability of the APP training (Figure 1A). A Decision Forest method was used for the classification. Additional post-processing steps were added to the APP as follows: Filling the holes—the area of DAB-positive cells, the area of tumor cells, and the area of the background was filled with itself (Figure 1B,C).

Output values are given as a percentage of the area of DAB-positive cells (CD3 or CD8) per the area (whole-tissue area randomly selected at 5× magnification) of tumor and stroma, respectively. As the platform could not distinguish between CD4-positive T cells and other CD4-positive cells (in the case of macrophage presence), the percentage of CD4-positive cells was calculated by subtracting the percentage of CD8-positive cells from the total percentage of CD3-positive cells.

### 2.6. Inflammation Status Calculation

For the inflammation status, *SII* and *PIV* were calculated to assess the interactions between inflammatory pro-cancer populations (neutrophils, platelets, and monocytes) and anticancer immune populations (lymphocytes), and were determined as follows:

$$SII=\frac{P\times N}{L}$$

$$PIV=\frac{P\times N\times M}{L}$$

where *P*, *N*, *M*, and *L* represent the absolute counts of platelets, neutrophils, monocytes, and lymphocytes, respectively, in milliliters of peripheral blood. These calculations were based on the methodology defined by Shang et al. [23]. The values for CA 125 (U/mL), LDH (U/L), and CRP (mg/L) were directly retrieved from laboratory data in the patient’s medical record, specifically from hemogram and biochemical analyses of patient’s blood samples, collected at the time of diagnosis.

### 2.7. Statistical Analysis

To achieve 80% statistical power to detect a hazard ratio (HR) of 2.0, with a two-sided α of 0.05 and an expected 50% event rate between low and high TIL groups, we estimated a required sample size of 56 patients, increasing to 58 to consider potential imbalance between groups. Descriptive analysis was performed to summarize the clinical characteristics of the patients. Intra-rater agreement for the manual assessment of TILs among both investigators (S.F.G. and S.M.) was examined with the interclass correlation coefficient (ICC). The ICC was described as very weak (0.00–0.19), weak (0.20–0.39), moderate (0.40–0.59), strong (0.60–0.79), and very strong (0.80–1.0). The association and correlation between the manual assessment of TILs and digital image analysis results were evaluated using Bland–Altman and Spearman’s tests, respectively. Optimal cut-off values for stratifying sTIL subsets and inflammation status (SII, PIV, CA 125, LDH, CRP) into low and high groups were determined using ROC curve analysis, selecting the values that maximized both sensitivity and specificity. The Area Under the Curve (AUC) was calculated for each ROC curve to evaluate diagnostic performance, with values interpreted as fail (<0.60), poor (0.60–0.69), fair (0.70–0.79), good (0.80–0.89), and excellent (0.90–1.00). The association between these stratified groups and PFS was analyzed using Kaplan–Meier curves. The association between TILs and immunoinflammatory biomarkers was assessed by chi-square testing. Additionally, univariate and multivariate analyses were performed to further evaluate the relationship between these parameters and PFS, with HRs and 95% confidence intervals (95% CIs) reported. The “10 events per variable” rule was applied to guide the multivariable Cox regression analysis. Statistical analysis was performed with SPSS v. 28.0.1.0 and GraphPrism v. 10.4.1.

## 3. Results

### 3.1. Patients’ Characteristics

**Figure 2:**
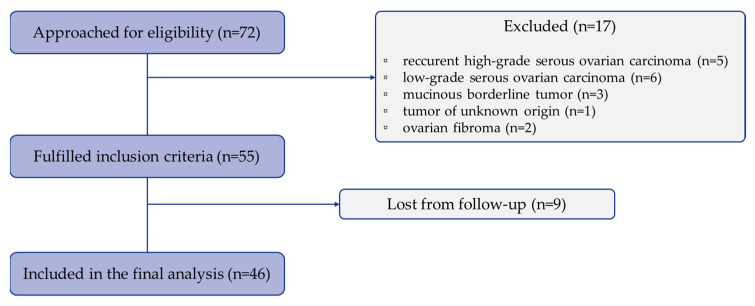
Flow chart of patients’ eligibility and final inclusion in the study.

Of a total of 72 patients initially considered eligible for inclusion in the study, after histological confirmation of the diagnosis, 5 patients were diagnosed with recurrent HGSC, 6 with low-grade serous ovarian carcinoma, 3 patients had mucinous borderline tumors, 1 patient had a tumor of unknown origin, and 2 patients had fibromas. As a result, 55 patients met the inclusion criteria. However, due to loss of follow-up data for 9 patients who continued treatment outside the OIL and UMCL, only 46 patients with histologically confirmed primary HGSC were included in the study and considered for further analysis (Figure 2).

The median age at diagnosis of the 46 patients included in the study was 64 years (range: 44–86 years). Based on the International Federation of Gynecology and Obstetrics (FIGO) staging system, 2 patients were diagnosed at stage II, 34 at stage III, and 10 at stage IV. A positive family history for HFSC was present in 17/46 (36.9%) patients, while 2/46 (4.3%) patients had a previous history of breast cancer. Ascites was present in all patients.

Of the total, 15 patients underwent primary surgery (and adjuvant chemotherapy), while 25 underwent interval surgery and 6 had inoperable disease (hence 31 patients received neoadjuvant chemotherapy). Within the 5-year follow-up of our data analysis, 39/46 patients (84.8%) experienced disease progression, with a median progression-free survival (PFS) of 22.31 months (range: 3.15–55.0 months). Meanwhile, 31/46 patients (61.4%) passed away, with a median overall survival (OS) of 35.52 months (range: 5.03–61.06 months). The clinical characteristics of the patients are presented in Table 1.

### 3.2. Tumor-Infiltrating Lymphocytes

**Figure 3:**
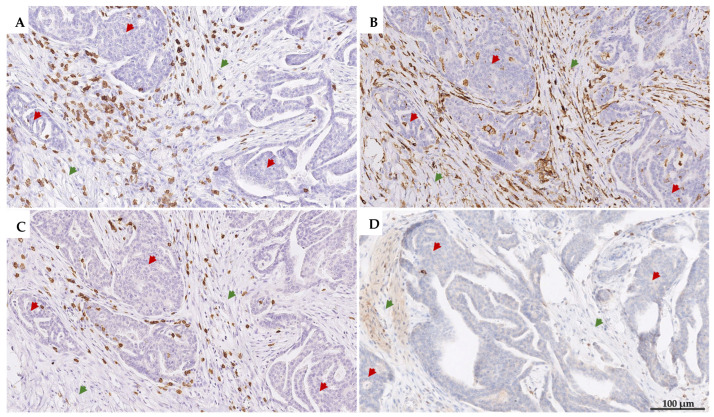
Representative images of (**A**) CD3^+^, (**B**) CD4^+^, (**C**) CD8^+^, and (**D**) PD-1+ iTILs (brown-stained cells) within the tumor epithelium (red arrows) and sTILs in the tumor stroma (green arrows). For CD4^+^, most positive cells are macrophages rather than lymphocytes, as evident from their morphology. Images are taken at 100× magnification.

**Figure 4:**
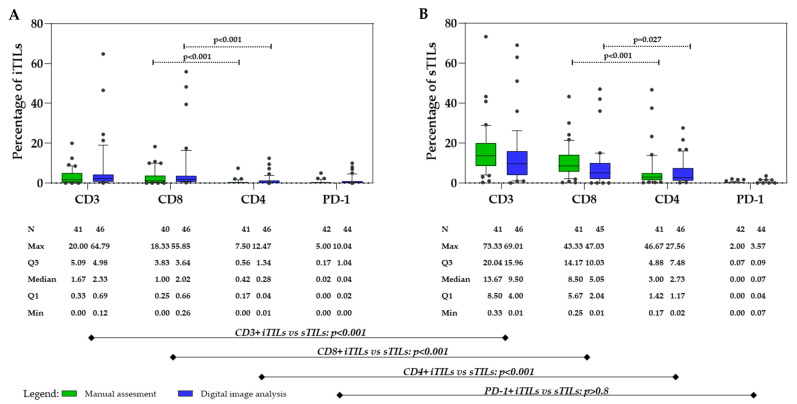
Box plots illustrating the median, minimum, maximum, and interquartile range (Q1–Q3) for CD3^+^, CD8^+^, CD4^+^, and PD-1+ (**A**) intratumoral tumor-infiltrating lymphocytes (iTILs) and (**B**) stromal tumor-infiltrating lymphocytes (sTILs), assessed manually (green) and by digital image analysis (blue). Note: the lines under the graphs show the correlation of the total count of CD3, CD8, CD4, and PD-1-iTILs (left graph) vs. their corresponding sTILs (right graph). Abbreviations: Min, minimum; Max, maximum; N, number of patients; Q1, first quartile; Q3, third quartile.

TILs were separately assessed as CD3^+^, CD4^+^, and CD8^+^ iTILs and sTILs, as shown in Figure 3. For manual assessment, two independent investigators (S.F.G. and S.M.) evaluated TILs, demonstrating excellent agreement (ICC = 0.968, 95% CI = 0.964–0.973, *p* < 0.001). Consequently, the average score of both investigators was used for further analysis of the results of the manual assessment of TILs (data on the individual assessment scores of each investigator are provided in Supplementary Table S1). According to the average score, the median percentage of iTILs was 1.67% (range: 0.00–20.00%), with CD8^+^ iTILs (median: 1.00%, range: 0.00–18.33%) predominating (*p* < 0.001) over CD4^+^ iTILs (median: 0.42%, range: 0.00–7.50%), while the median percentage of PD-1+ iTILs was very low (median: 0.02%, range: 0.00–5.00%) (Figure 4A).

The percentage of CD3^+^, CD4^+^, and CD8^+^ sTILs was significantly higher than the corresponding iTIL subsets (*p* < 0.001) (Figure 4B). The median percentage for CD3^+^ sTILs was 13.67% (range: 0.33–73.33%), while the median percentage for CD8^+^ sTILs was 8.50% (range: 0.25–43.33%) and for CD4^+^ it was sTILs 3.00% (range: 0.17–46.67%). CD8^+^ sTILs were significantly more abundant than CD4^+^ sTILs (*p* < 0.001), while the median percentage of PD-1+ sTILs was 0.00% (range: 0.00–2.00%), similar to PD-1+ iTILs, which were detected in 9/41 cases (21.95%).

According to the results of the digital image analysis (Figure 4A,B) of the same iTIL and sTIL subsets, the median CD3^+^ iTIL percentage was 2.33% (range: 0.12–64.79%), and the median CD8^+^ iTILs percentage was 2.02% (range: 0.26–55.85%). The median of CD4 iTILs was calculated by subtracting CD8^+^ from CD3^+^ iTILs, which resulted to 0.28% (range 0.01–12.47%), indicating predominance of CD8^+^ iTILs over CD4^+^ iTILs (*p* < 0.001). The median percentage of PD-1+ iTIL was very low, at 0.04 (0.00–10.04%). Significantly higher percentages of sTILs were observed compared to iTILs (*p* < 0.001), except for PD-1+ TILs (*p* > 0.8). The median percentage of CD3^+^ sTILs was 15.96% (range: 0.01–69.01%), with CD8^+^ sTILs (median: 5.05%, range: 0.01–47.03%) prevailing (*p* = 0.027) over CD4^+^ sTILs (median: 2.73%, range: 0.02–27.56%), which were calculated by subtracting CD8^+^ from CD3^+^ sTILs. The median percentage of PD-1+ sTILs was as low as that of PD-1+ iTILs (median: 0.07%, range: 0.02–3.57%).

**Figure 5:**
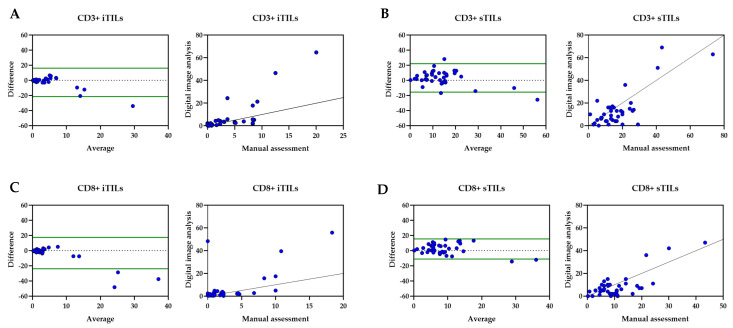
The association and correlation between manual and digital image analysis assessed by Bland–Altman dot plot (left) and Spearman ‘s rank dot plot (right) for (**A**) CD3^+^ iTILs, (**B**) CD3^+^ sTILs, (**C**) CD8^+^ iTILs, and (**D**) CD8 sTILs.

The statistical comparison of both methods for iTIL and sTIL subsets revealed very strong association and correlation between the two methods, showing consistent results and patterns (Figure 5).

### 3.3. Inflammation Status

The analysis of inflammation status at diagnosis showed a median SII value of 1466.27 (range: 89.62–7397.29) and a median PIV value of 919.98 (range: 143.90–5695.91). The median CA 125 was 168.50 U/mL (range: 10.27–22,531.00 U/mL), with a reference value below 35 U/mL. The median LDH was 3.2 µkat/L (range: 2.22–36.79 µkat/L), with a reference value below 4.12 µkat/L. The median CRP was 6.9 mg/L (range: 0.7–169.70 mg/L), with a reference value below 5 mg/L.

### 3.4. ROC Curve Analysis

To classify the percentages of TILs and values of the immune-inflammatory biomarkers from continuous variables as a binary outcome (low vs. high or yes vs. no), a ROC curve analysis was performed (Table 2, Supplementary Figure S1).

The optimal cut-off values for manually assessed sTILs were 12.9% for CD3^+^, 5.66% for CD8^+^, and 1.83% for CD4^+^. For sTILs assessed using digital image analysis, the cut-off values were 4.49% for CD3^+^ and 2.03% for CD8^+^ cells. The cut-off for CD4^+^ sTILs was not further assessed, as CD4^+^ cells were approximately estimated by subtracting CD8^+^ from CD3^+^ sTILs. Additionally, ROC curve analysis and any further analysis of the iTIL subset were not performed due to their very low percentages, which prevented the calculation of reliable results.

The optimal cut-off values for the immune-inflammatory biomarkers were determined as follows: 912.45 × 10^9^/L for SII, 423.92 × 10^9^/L for PIV, 64.5 U/mL for CA 125, 3.01 µkat/L for LDH, and 6.55 mg/L for CRP. Additionally, for the FIGO staging system, stages II and III were grouped together and compared to stage IV.

### 3.5. Univariate Analysis

**Figure 6:**
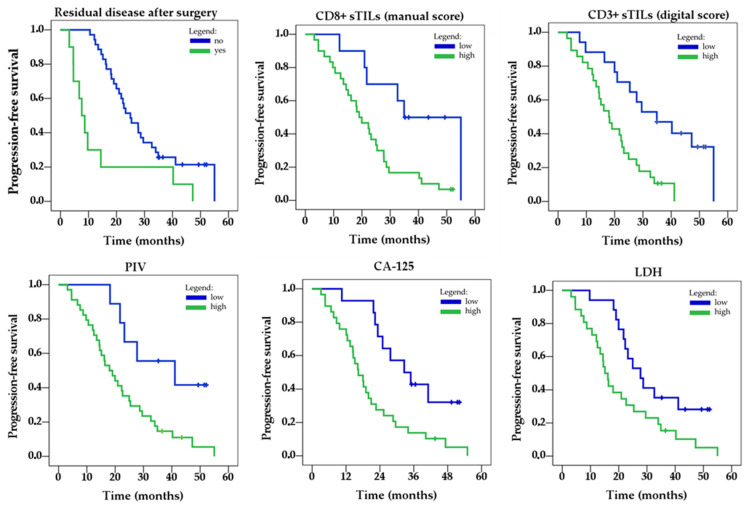
Kaplan–Meier curves for the significant clinical parameters and TILs identified in the univariate analysis.

Following the ROC curve analysis, a univariate analysis was performed to assess the association of sTILs and immune-inflammatory biomarkers with patients’ PFS. The results of the univariate analysis are summarized in Table 3 and Figure 6.

The univariate analysis revealed that residual disease after surgery (no vs. yes) showed significant associations with PFS, with HRs of 0.34 (95% CI: 0.16–0.72, *p* = 0.005), while FIGO stage (II and III) against IV did not reach statistical significance.

Among sTILs, manually assessed CD8^+^ and digitally assessed CD3^+^ showed a significant association with PFS, with HRs of 0.30 (95% CI: 0.12–0.79, *p* = 0.015) and 0.31 (95% CI: 0.15–0.97, *p* = 0.003), respectively. In contrast, manually assessed CD3^+^ sTILs and digitally assessed CD8^+^ sTILs did not reach statistical significance, although they showed a trend toward significance, with *p*-values of 0.064 and 0.066, respectively.

PIV (low vs. high) was significantly associated with PFS, with an HR of 0.32 (95% CI: 0.12–0.82, *p* = 0.018), while SII (low vs. high) did not reach statistical significance (HR: 0.51, 95% CI: 0.24–1.09, *p* = 0.080). CA 125 (low vs. high) and LDH (low vs. high) were also significantly associated with PFS, with HRs of 0.35 (95% CI: 0.16–0.75, *p* = 0.007) and 0.44 (95% CI: 0.22–0.89, *p* = 0.022), respectively. CRP (low vs. high) did not reach statistical significance (HR: 0.56, 95% CI: 0.29–1.09, *p* = 0.090).

CD8^+^ sTILs, the only manually assessed TIL subtype with significant prognostic value in the cohort, was further analyzed for associations with clinical parameters (FIGO stage, residual disease) and inflammation status (SII, PIV, CA 125, LDH, CRP) using chi-square tests. However, no significant associations were found, although residual disease and CRP demonstrated a trend toward significance (*p* = 0.083 and *p* = 0.065), as shown in Supplementary Table S2.

### 3.6. Multivariate Analysis

In the multivariate analysis, two models were tested, one including manual evaluation of sTILs and one including digital image analysis of TILs. Following the rule of 10 events for each factor studied, only three variables that were statistically significant in the univariate analysis were included in the model. In both models, PIV at diagnosis, residual disease after surgery, and sTILs (CD8^+^ sTIL for manual and CD3^+^ sTIL for digital image assessment) were used.

In the multivariate model containing manually assessed sTILs, low PIV (HR = 0.32, 95% CI: 0.11–0.91, *p* = 0.032) and low CD8^+^ sTILs (HR = 0.30, 95% CI: 0.11–0.84, *p* = 0.021) were independent favorable prognostic factors for PFS. However, in the multivariate model with digitally assessed sTILs, all three parameters were independent favorable prognostic factors: low PIV (HR = 0.35, 95% CI: 0.13–0.96, *p* = 0.040), no residual disease after surgery (HR = 0.21, 95% CI: 0.08–0.53, *p* = 0.001), and low CD3^+^ sTILs (HR = 0.16, 95% CI: 0.06–0.42, *p* < 0.001). The results of the multivariate analysis are shown in Table 4.

## 4. Discussion

In this study, we assessed the association between TILs and inflammation status in patients with primary HGSC, and their correlation with PFS. We provided a detailed characterization of CD3^+^, CD4^+^, CD8^+^, and PD-1+ in percentages of iTILs and sTILs in primary HGSC, assessed at the time of diagnosis and in accordance with TIL-WG recommendations, using both manual and digital image analysis. Our results offered insight into the distribution of TIL subtypes among patients and highlighted digital image analysis as a promising tool for reliable TIL assessment in the near future. In parallel, we evaluated systemic inflammation status and found that low PIV, CA 125, and LDH were associated with better PFS, while CD3^+^ and CD8^+^ sTILs, along with PIV, emerged as potential independent prognostic biomarkers.

These findings are particularly relevant given that HGSC is the most lethal form of ovarian cancer, yet effective prognostic markers remain limited. In recent years, the tumor-immune microenvironment has emerged as a promising area for identifying novel biomarkers that could improve patient stratification and outcome prediction. Both TILs and inflammation score have attracted considerable research attention [24]. However, despite encouraging findings, results across studies remain inconsistent for both TILs and inflammatory markers in HGSC. A major contributing factor is that much of the published data combine different histological subtypes and disease stages, which can significantly influence outcomes and limit the specificity of prognostic conclusions. In this context, our study focused exclusively on primary HGSC at the time of diagnosis, investigating the association of TILs (both iTILs and sTILs) and inflammation score (SII, PIV, CA 125, LDH, and CRP) with progression-free survival.

TILs, in particular, have demonstrated potential as prognostic markers in HGSC, reflecting the strength of the anti-tumor immune response and frequently correlating with improved survival outcomes [4,5,6,7,9,11]. However, discrepancies in reported findings persist. A significant source of this variability lies in inconsistent methodologies used to evaluate TILs. Published studies differ in whether they assess iTILs, sTILs, or a combination, and apply a range of methodological approaches, from microarrays focused on tumor hotspots to whole-tissue sections analyzed at 200× or 400× magnification, using varying numbers of HPFs. The assessment approach also varies, with some studies using H&E staining and others relying on IHC, while scoring systems range from manually applied criteria to internally developed digital algorithms [7,25]. To address these inconsistencies, the TIL-WG proposed standardized guidelines for manual scoring of iTILs and sTILs separately, which involve estimating the percentage of tumor-associated stroma occupied by iTILs or sTILs, while excluding necrosis and normal tissue [12,13]. Since 2019, the clinical relevance of sTILs has been acknowledged by major organizations such as the St. Gallen Consensus, World Health Organization (WHO), and European Society of Medical Oncology (ESMO) [26]. Nonetheless, not all published studies on ovarian carcinoma, particularly HGSC, have adopted these guidelines in practice. The TIL-WG has also emphasized the importance of computational assessment of sTILs to overcome current limitations, recommending that digital algorithms follow manual scoring principles where appropriate [12].

In our study, TILs were assessed according to the standardized recommendations of the TIL-WG [12,13]. We achieved excellent intra-rater agreement among both investigators during manual evaluation. This consistency confirms that results of our TIL quantification are reliable, which is essential before performing any further analysis to avoid misleading conclusions. Further, we incorporated digital image analysis, which showed a strong correlation with manual scoring. Digital image analysis in our study was primarily employed to assess whether it could reproduce the same results as manual counting when appropriately trained. The strong correlation we observed between the two methods highlights the potential of digital image analysis as a promising tool for future standardized and automated TIL assessment.

Based on the confirmed reliability of our evaluation score of TILs, an important contribution of our study is the detailed characterization of median percentages of CD3^+^, CD4^+^, CD8^+^, and PD-1+ iTILs and sTILs in primary HGSC at the time of diagnosis. The only comparable datapoint we identified was the overall mean sTIL percentage reported by Hwang et al. for serous ovarian carcinoma (8.06%), which was slightly lower than our findings of 13.67% by manual assessment and 9.50% by digital image analysis [12]. However, to our knowledge, no other study has reported separately stratified percentage data for CD3^+^, CD4^+^, and CD8^+^ TILs across both stromal and intraepithelial compartments.

Our results demonstrated that the percentages of iTILs were significantly lower than those of sTILs across all evaluated subsets (Figure 1A,B). An exception was observed for PD-1+ TILs, which showed very low percentages in both iTILs and sTILs, consistently below 1%. Furthermore, CD8^+^ TILs were significantly more abundant than CD4^+^ TILs within both iTILs and sTILs. Interestingly, this contrasts with findings from our previous study on ascitic fluid from the same patient cohort, where CD4^+^ T cells were more prevalent than CD8^+^ T cells, although the difference was not statistically significant [27]. Moreover, the levels of CD3^+^, CD4^+^, CD8^+^, and PD-1+ lymphocytes in ascitic fluid were substantially higher than the corresponding TILs in primary tumor tissue from our previous study, with values exceeding 40% for all markers except PD-1+, for which the value was around 20% [27]. Similar results were also observed by Bekos et al., who compared immune cell profiles between primary ovarian tumors and metastatic sites and reported notable differences in CD8^+^ and PD-1+ T-cell distributions [6]. These observations suggest that immune evasion may vary by tumor or metastatic site, highlighting the need to consider spatial immune heterogeneity when developing predictive biomarkers for potential immunotherapy in the near future. Additionally, we would like to point out that we detected very low levels of PD-1+ iTILs and sTILs in our cohort, consistently below 1%, which was consistent with our previous study on PD-1 in spheroids in the ascites of HSGC patients [28]. We did not find literature reporting exact percentage values for PD-1+ TILs, as most studies classify PD-1 expression qualitatively. However, based on the representative published IHC images and the classification of patients into high and low PD-1+ TIL groups, we infer that studies such as those by Drakes et al., Darb-Esfahani et al., and De la Fuente et al. reported higher PD-1 expression levels than those observed in our cohort [29,30,31].

Returning to the assessment criteria of TILs, which are crucial for stratifying patients into low and high TIL groups when analyzing correlations with clinical outcomes, it is worth noting that although most studies follow this approach, no standardized cut-off has been established. As a result, a wide range of cut-off values has been reported so far. For instance, James et al. used a classification of very low (1%), low (5%), and high (≥10%), while Taangard applied a fixed cut-off of 10% TILs [8,32]. Some authors, such as Sato et al., have used the median TIL value within their cohort as a threshold between low and high values, whereas others have applied arbitrarily defined cut-offs, often adapted from breast cancer studies or based on internally set criteria [4,8,33,34]. In contrast, our study employed ROC curve analysis to define an optimal cut-off point. ROC analysis provides a robust, objective way to define cut-offs by maximizing sensitivity and specificity while enabling statistical significance of their prognostic value. Dai et al. similarly used ROC curve analysis to determine cut-offs in their study, reinforcing its appropriateness in the context of HGSC [35]. It is worth noting that some studies quantified TILs by the number of cells per area, for example, using categories such as low (1–2 cells), moderate (3–10 cells), and high (≥20 cells) as proposed by Stanske et al.; low/moderate (1–19 cells) vs. high (>20 cells), as in the OTTA Consortium and Pizarro et al.; or semi-quantitative scoring systems like + (1–25 cells), ++ (25–50 cells), and +++ (>50 cells), applied by Raltore et al. and Gomez-Marcia et al. [9,10,36,37,38].

Regardless of the differences in cut-off values used to define low, medium, and high TIL levels, the literature consistently suggests that higher infiltration of CD3^+^ and CD8^+^ iTILs and sTILs is significantly associated with longer patient survival, as demonstrated in both univariate and multivariate analyses [4,7]. Notably, both studies assessed TILs in cohorts comprising EOC cases overall, not limited exclusively to HGSC. On the other hand, some studies, such as Alyeva et al., did not confirm a statistically significant relationship between CD8^+^ TIL subsets and patient survival in a cohort of 45 EOC patients—neither before nor after neoadjuvant chemotherapy [39].

Our results showed that high levels of CD3^+^ and CD8^+^ sTILs were associated with worse patient survival, which contrasts with the majority of published data. To our knowledge, only one study, performed by Karakaya et al., supports our findings [40]. One possible explanation for this discrepancy is the presence of exhausted T cells that have lost their cytotoxic function due to chronic antigen stimulation, and consequently upregulate inhibitory receptors such as PD-1, T-cell immunoglobulin, mucin-domain containing-3 (TIM-3), and lymphocyte activation gene-3 (LAG-3). Exhausted T cells are characterized by impaired cytokine production, diminished persistence, and distinct transcriptional and epigenetic profiles. Other contributing factors may include immunosuppressive cytokines (e.g., interleukin-10, TGF-β), metabolic stress within the tumor microenvironment, hormonal influences, and insufficient CD4^+^ T-cell help during early priming. Although we evaluated PD-1 expression, the detected percentages were very low, preventing any meaningful correlation with patient survival. Given that PD-1 is a well-established marker of T-cell exhaustion, and its upregulation is typically observed in exhausted T cells, we would have expected higher levels of PD-1 expression in our cohort. We speculate that these unexpectedly low values may be attributable to methodological differences in our PD-1 IHC protocol rather than the antibody clone itself. We used the NAT-105 clone, which is among the most commonly used in published studies according to the reviewed literature [31,40,41,42]. Our technical limitation warrants further investigation. Future studies should focus on optimizing the PD-1 IHC protocol and expanding the analysis to include additional T-cell exhaustion markers, such as TIM-3 and LAG-3. The exhausted status of CD8^+^ TILs can be more accurately assessed through the co-expression of PD-1 and TIM-3, which has been associated with poor prognosis, as suggested by Sewada et al. [43]

Alternatively, the CD8^+^ T cells in our cohort may represent the Tc1 subtype. While generally cytotoxic, Tc1 cells produce cytokines such as interferon-γ (IFN-γ), tumor necrosis factor-α (TNF-α), and granzyme B, which may exert immunomodulatory effects and, under certain conditions, support tumor progression through mechanisms that resemble those of CD4^+^ helper T cells [44]. Including granzyme B in future analyses could help further characterize the presence and functional state of Tc1 cells. Such investigations will also help understand the mechanisms underlying our findings.

Several studies have shown that CD4^+^ TILs are associated with improved outcomes in ovarian cancer, likely due to their role in recruiting and activating CD8^+^ T cells, although others have reported no association or even a negative impact on prognosis [25,45,46,47]. In our analysis, CD4^+^ TILs were not associated with patient survival, which may reflect the functional heterogeneity of CD4^+^ subsets [25]. Similarly, PD-1 expression on CD8^+^ TILs has been linked to favorable clinical outcomes in ovarian cancer, yet we did not observe such an association in our cohort. This discrepancy may be related to the overall lower PD-1 expression levels we observed compared to others.

Considering that tumor growth and progression result from a reciprocal interplay between cancer cells and host immune cells, where both innate and adaptive mechanisms shape a tumor-promoting and immunosuppressive microenvironment, the role of systemic inflammation in cancer progression should not be overlooked [48,49]. Inflammation status scored by SII and PIV has become the focus of increasing research as a prognostic marker in various malignancies, including ovarian carcinoma. SII is calculated from platelet, neutrophil, and lymphocyte counts, while PIV includes monocytes as an additional component [25]. Although several studies have shown that elevated SII is associated with worse PFS and OS [49,50,51], there are also studies, including ours, that did not reach statistical significance for the predictive value of SII. This variability may be due to differing cut-off values across studies, ranging from 564.8 to 1000 (912.5 in our cohort), and the inclusion of various epithelial ovarian cancer subtypes [50]. PIV is a relatively new biomarker, studied mainly in colorectal and breast cancers, where it has shown stronger predictive value than SII [19], as we also confirmed in this study. A meta-analysis by Kuang et al. linked elevated PIV to poorer PFS and OS across several cancer types [52]. Although less studied in ovarian cancer, a retrospective study by Liao et al. of 576 patients confirmed its prognostic value, aligning with our findings [19]. Considering that lymphocytes are the denominator in both the SII and PIV formulas, and that higher levels of CD3 and CD8 sTILs in our study were associated with worse PFS, elevated PIV may reflect a systemic environment characterized by increased neutrophil and monocyte counts, both associated with immunosuppressive effects, and reduced lymphocyte levels. This may promote conditions in which immune effector cells such as CD8^+^ TILs become functionally impaired.

Additionally, in many studies, elevated levels of CA 125, LDH, and CRP have been associated with worse progression-free and overall survival. CA 125 is the most extensively studied and validated biomarker in epithelial ovarian cancer and is routinely used to monitor patients diagnosed and treated for HGSC. At initial diagnosis, CA 125 levels (normal range <35 U/mL) are elevated in approximately 80% of epithelial ovarian cancer patients and correlate well with treatment response, disease progression, and recurrence [53,54]. Higher CA 125 levels are generally observed in more advanced disease stages. In line with our findings, Ay et al. also confirmed the statistically significant predictive value of initial CA 125 levels [55]. Interestingly, one study by Asali et al. investigated advanced-stage epithelial ovarian carcinoma patients with low CA 125 levels and found that they had similar clinical outcomes to patients with elevated CA 125, suggesting that even low levels do not preclude aggressive disease behavior [56].

LDH, although primarily used in clinical settings to detect tissue damage in conditions such as myocardial infarction, hepatitis, or hemolysis, is also elevated in malignant conditions due to tumor proliferation and metabolic stress. In ovarian cancer, LDH has been shown to correlate with disease progression and worse survival outcomes [57]. Bastani et al. further indicated that LDH may help distinguish malignant from benign ovarian tumors [58]. As with CA 125, LDH levels tend to increase with advancing clinical stage and histological grade.

CRP has also been explored as a prognostic marker in ovarian cancer. Hefler et al. reported that CRP levels above 3.6 mg/L were significantly associated with advanced FIGO stage, poor overall survival, and platinum resistance [21]. Yang et al. similarly found that CRP levels above 9.8 mg/L correlated with worse survival [59]. In our study, the cut-off CRP level was 6.55 mg/L and, although not statistically significant, it showed a trend toward association with survival outcomes. This difference may reflect variability in cohort characteristics or sample size. Nonetheless, combining CA 125 with other inflammation scores such as LDH and CRP has been shown to enhance diagnostic and prognostic assessment in ovarian cancer.

However, our study had some limitations. One of the main limitations of our study was the small final cohort of 46 patients, which reduced the statistical power, even though 58 patients were calculated to be necessary to achieve 80% power. Although 72 patients were initially approached within the study duration, more than one-third met the exclusion criteria due to non-relevant histological subtypes, recurrent HGSC, or insufficient follow-up data, ultimately reducing the number of eligible cases and limiting the multivariable analysis to three covariates. Nevertheless, in our univariate analyses, CD8^+^ sTILs were significant only in the manual assessment, whereas CD3^+^ sTILs reached significance only in the digital evaluation; neither marker was significant across both methods, although each showed a consistent trend in the respective alternate approach. This highlights the importance of patient number in detecting robust associations. However, in two multivariate models combining sTILs, PIV, and residual disease after surgery, both CD8^+^ (manual assessment) and CD3^+^ (digital image analysis) remained potential independent predictors of progression-free survival alongside PIV. Further validation, particularly of CD8^+^ and CD3^+^ sTILs, is essential in larger, ideally multicentered studies. The limited sample size may also explain why we did not observe a potential significant association for SII and CRP, or detect correlations between TILs and inflammation status scored by PIV, SII, CA 125, LDH, and CRP, which should be also re-evaluated in studies with greater statistical power. Another limitation important to mention is that digital image analysis was performed using an older version of VisioPharm software (2021.02). Since then, multiple upgrades have been released, culminating in the most recent version (2025.02.1) in March 2025. At that time, the analysis process was more time-consuming, particularly for algorithm training and result generation. Since then, the software has been continuously improved, with enhanced capabilities for recognizing IHC reactions and interpreting cell morphology, making newer versions more efficient and even more user-friendly. Despite all these limitations, we believe our exploratory findings are hypothesis-generating and can stimulate further investigation.

## 5. Conclusions

In conclusion, this is the first study to provide a percentage-based profile of CD3^+^, CD4^+^, CD8^+^, and PD-1+ iTILs and sTILs within the same cohort of patients with primary HGSC at the time of diagnosis, following the TIL-WG recommendations, and using both manual and digital image analysis. By evaluating these subsets concurrently, our study offers a direct comparative overview of TIL distribution across compartments, enhancing the informative value of the data. We observed a consistent dominance of CD8^+^ over CD4^+^ T cells and very low PD-1+ expression in both stromal and intraepithelial regions. In parallel, we assessed inflammation status scored by SII, PIV, CA 125, LDH, and CRP. Although no significant associations were observed between TILs and inflammation status, both CD3^+^ (digital image analysis) and CD8^+^ sTILs (manual assessment) and PIV emerged as potential independent predictors of PFS in the multivariate analysis, suggesting possible prognostic utility. Still, due to the limited cohort size, larger studies are needed to extend the multivariate analysis and further investigate the potential significance of trends observed in non-significant markers in our study.

## Figures and Tables

**Table 1:** Clinical characteristics of the patients with primary high-grade serous carcinoma included in the study.

Clinical Characteristics	
** Age at diagnosis, years**	
*Mean (range)*	64 (44–86)
**FIGO stage, No. (%)**	
*II*	2 (4.3)
*IIIA*	1 (2.2)
*IIIB*	1 (2.2)
*IIIC*	32 (69.6)
*IVA*	4 (8.7)
*IVB*	6 (13.0)
**Positive family history, No. (%)**	
*breast*, *ovary*, *prostate*, *pancreas*	17 (36.9)
**Chemotherapy, No. (%)**	
*neoadjuvant*	31 (67.4)
*adjuvant*	15 (32.6)
**Surgery, No. (%)**	
*Primary*	15 (32.6)
*Interval*	25 (54.4)
*Inoperable*	6 (13.0)
**Patient status, No. (%)**	
*Progression*	39 (84.8)
*Death*	31 (61.4)
**Ascites presence, No. (%)**	
yes	46 (100%)

**Table 2:** ROC curve analysis of clinical parameters and TILs determined at the time of diagnosis in the analyzed patient cohort. The significant *p*-values are shown in bold.

Parameter at Diagnosis	Cut-Off Value by ROC Curve	AUC	CI 95%	*p*-Value	Sensitivity	Specificity
CD3^+^ sTILs manual assessment, low vs. high	12.9%	0.645	0.474–0.816	0.261	0.657	0.667
CD8^+^ sTILs manual assessment, low vs. high	5.66%	0.731	0.508–0.954	0.074	0.829	0.667
CD4^+^ sTILs manual assessment, low vs. high	1.83%	0.764	0.615–0.913	0.410	0.714	0.667s
CD3^+^ sTILs digital image analysis, low vs. high	4.49%	0.736	0.525–0.948	**0.049**	0.821	0.714
CD8^+^ sTILs digital image analysis, low vs. high	2.03%	0.748	0.565–0.931	**0.039**	0.816	0.714
SII low vs. high	912.45 × 10^9^/L	0.715	0.464–0.966	0.094	0.763	0.667
PIV low vs. high	423.92 × 10^9^/L	0.811	0.575–1.000	**0.015**	0.868	0.667
CA 125 low vs. high	64.5 U/mL	0.768	0.560–0.975	0.370	0.737	0.833
LDH low vs. high	3.01 µkat/L	0.761	0.495–1.000	**0.042**	0.658	0.833
CRP low vs. high	6.55 mg/L	0.724	0.495–0.952	0.081	0.579	0.833

**Table 3:** Univariate analysis of the clinical parameters, TILs, immune-inflammatory environment, and PFS. The significant *p*-values are shown in bold.

Parameter at Diagnosis	Hazard Ratio	CI 95%	*p*-Value
FIGO stage II+III vs. stage IV	0.66	0.32–1.37	0.266
Residual disease after surgery no vs. yes	0.34	0.16–0.72	**0.005**
CD3^+^ sTILs manual score low vs. high	0.50	0.24–1.04	0.064
CD8^+^ sTILs manual score low vs. high	0.30	0.12–0.79	**0.015**
CD3^+^ sTILs digital score low vs. high	0.31	1.15–0.67	**0.003**
CD8^+^ sTILs digital score low vs. high	0.53	0.27–1.04	0.066
PIV low vs. high	0.32	0.12–0.82	**0.018**
SII low vs. high	0.51	0.24–1.09	0.080
CA 125 low vs. high	0.35	0.16–0.75	**0.007**
LDH low vs. high	0.44	0.22–0.89	**0.022**
CRP low vs. high	0.56	0.29–1.09	0.090

**Table 4:** Multivariate analysis of the significant clinical parameters and TILs from the univariate analysis for PFS. The significant *p*-values are shown in bold.

Model	Variable	Hazard Ratio	CI 95%	*p*-Value
*Model 1: Manual assessment of sTILs*				
	PIV low vs. high	0.32	0.11–0.91	**0.032**
	Residual disease after surgery no vs. yes	0.81	0.33–2.00	0.649
	CD8^+^ sTILs (manual score) low vs. high	0.30	0.11–0.84	**0.021**
*Model 2: Digital image analysis of sTILs*				
	PIV low vs. high	0.35	0.13–0.96	**0.04**
	Residual disease after surgery no vs. yes	0.21	0.08–0.53	**0.001**
	CD3^+^ sTILs (digital score) low vs. high	0.16	0.06–0.42	**<0.001**

## Data Availability

All relevant data regarding this manuscript are available from the above-listed authors.

## Supplementary Materials

The following supporting information can be downloaded at: https://www.mdpi.com/article/10.3390/cancers17142269/s1. Supplementary Table S1. Individual score of the manual assessment of TILs of both investigators (S.F.G. and S.M.). Supplementary Table S2. Association between CD8^+^ sTIL and the other clinical characteristics/inflammation status. Supplementary Figure S1. ROC curves for the analyzed parameters to determine the cut-off values for classifying low vs. high groups.

## References

[1] Torre L.A., Trabert B., DeSantis C.E., Miller K.D., Samimi G., Runowicz C.D., Gaudet M.M., Jemal A., Siegel R.L. (2018). Ovarian cancer statistics, 2018. CA A Cancer J. Clin..

[2] Jayde V., White K., Blomfield P. (2009). Symptoms and diagnostic delay in ovarian cancer: A summary of the literature. Contemp. Nurse.

[3] Zhang L., Conejo-Garcia J.R., Katsaros D., Gimotty P.A., Massobrio M., Regnani G., Makrigiannakis A., Gray H., Schlienger K., Liebman M.N. (2003). Intratumoral T cells, recurrence, and survival in epithelial ovarian cancer. N. Engl. J. Med..

[4] Sato E., Olson S.H., Ahn J., Bundy B., Nishikawa H., Qian F., Jungbluth A.A., Frosina D., Gnjatic S., Ambrosone C. (2005). Intraepithelial CD8^+^ tumor-infiltrating lymphocytes and a high CD8^+^/regulatory T cell ratio are associated with favorable prognosis in ovarian cancer. Proc. Natl. Acad. Sci. USA.

[5] Curiel T.J., Coukos G., Zou L., Alvarez X., Cheng P., Mottram P., Evdemon-Hogan M., Conejo-Garcia J.R., Zhang L., Burow M. (2004). Specific recruitment of regulatory T cells in ovarian carcinoma fosters immune privilege and predicts reduced survival. Nat. Med..

[6] Bekos C., Pils D., Dekan S., Hofstetter G., Horak P., Reinthaller A., Polterauer S., Schwameis R., Aust S. (2021). PD-1 and PD-L1 expression on TILs in peritoneal metastases compared to ovarian tumor tissues and its associations with clinical outcome. Sci. Rep..

[7] Hwang C., Lee S.J., Lee J.H., Kim K.H., Suh D.S., Kwon B.S., Choi K.U. (2019). Stromal tumor-infiltrating lymphocytes evaluated on H&E-stained slides are an independent prognostic factor in epithelial ovarian cancer and ovarian serous carcinoma. Oncol. Lett..

[8] James F.R., Jiminez-Linan M., Alsop J., Mack M., Song H., Brenton J.D., Pharoah P.D.P., Ali H.R. (2017). Association between tumour infiltrating lymphocytes, histotype and clinical outcome in epithelial ovarian cancer. BMC Cancer.

[9] Goode E.L., Block M.S., Kalli K.R., Vierkant R.A., Chen W., Fogarty Z.C., Gentry-Maharaj A., Tołoczko A., Hein A., Ovarian Tumor Tissue Analysis (OTTA) Consortium (2017). Dose-Response Association of CD8^+^ Tumor-Infiltrating Lymphocytes and Survival Time in High-Grade Serous Ovarian Cancer. JAMA Oncol..

[10] Stanske M., Wienert S., Castillo-Tong D.C., Kreuzinger C., Vergote I., Lambrechts S., Gabra H., Gourley C., Ganapathi R.N., Kolaschinski I. (2018). Dynamics of the Intratumoral Immune Response during Progression of High-Grade Serous Ovarian Cancer. Neoplasia.

[11] Fanale D., Dimino A., Pedone E., Brando C., Corsini L.R., Filorizzo C., Fiorino A., Lisanti M.C., Magrin L., Randazzo U. (2022). Prognostic and Predictive Role of Tumor-Infiltrating Lymphocytes (TILs) in Ovarian Cancer. Cancers.

[12] Salgado R., Denkert C., Demaria S., Sirtaine N., Klauschen F., Pruneri G., Wienert S., Van den Eynden G., Baehner F.L., Penault-Llorca F. (2015). The evaluation of tumor-infiltrating lymphocytes (TILs) in breast cancer: Recommendations by an International TILs Working Group 2014. Ann. Oncol. Off. J. Eur. Soc. Med. Oncol..

[13] Hendry S., Salgado R., Gevaert T., Russell P.A., John T., Thapa B., Christie M., Van De Vijver K., Estrada M.V., Gonzalez-Ericsson P.I. (2017). Assessing Tumor-infiltrating Lymphocytes in Solid Tumors: A Practical Review for Pathologists and Proposal for a Standardized Method From the International Immunooncology Biomarkers Working Group: Part 1: Assessing the Host Immune Response, TILs in Invasive Breast Carcinoma and Ductal Carcinoma In Situ, Metastatic Tumor Deposits and Areas for Further Research. Adv. Anat. Pathol..

[14] Laury A.R., Blom S., Ropponen T., Virtanen A., Carpén O.M. (2021). Artificial intelligence-based image analysis can predict outcome in high-grade serous carcinoma via histology alone. Sci. Rep..

[15] Farahani H., Boschman J., Farnell D., Darbandsari A., Zhang A., Ahmadvand P., Jones S.J.M., Huntsman D., Köbel M., Gilks C.B. (2022). Deep learning-based histotype diagnosis of ovarian carcinoma whole-slide pathology images. Mod. Pathol. Off. J. United States Can. Acad. Pathol..

[16] Machuca-Aguado J., Conde-Martín A.F., Alvarez-Muñoz A., Rodríguez-Zarco E., Polo-Velasco A., Rueda-Ramos A., Rendón-García R., Ríos-Martin J.J., Idoate M.A. (2023). Machine Learning Quantification of Intraepithelial Tumor-Infiltrating Lymphocytes as a Significant Prognostic Factor in High-Grade Serous Ovarian Carcinomas. Int. J. Mol. Sci..

[17] Song Q., Xu S.X., Wu J.Z., Ling L., Wang S., Shu X.H., Ying D.N., Pei W.W., Wu Y.C., Sun S.F. (2023). The preoperative platelet to neutrophil ratio and lymphocyte to monocyte ratio are superior prognostic indicators compared with other inflammatory biomarkers in ovarian cancer. Front. Immunol..

[18] Bizzarri N., D’Indinosante M., Marchetti C., Tudisco R., Turchiano F., Scambia G., Fagotti A. (2023). The prognostic role of systemic inflammatory markers in apparent early-stage ovarian cancer. Int. J. Clin. Oncol..

[19] Liao W., Li J., Feng W., Kong W., Shen Y., Chen Z., Yang H. (2024). Pan-immune-inflammation value: A new prognostic index in epithelial ovarian cancer. BMC Cancer.

[20] Deeba F., Khatun S., Alam M.M., Shahida S.M. (2015). Serum LDH and CA-125: Markers for Diagnosis of Ovarian Malignancy. Mymensingh Med. J. MMJ.

[21] Hefler L.A., Concin N., Hofstetter G., Marth C., Mustea A., Sehouli J., Zeillinger R., Leipold H., Lass H., Grimm C. (2008). Serum C-reactive protein as independent prognostic variable in patients with ovarian cancer. Clin. Cancer Res. Off. J. Am. Assoc. Cancer Res..

[22] Ledermann J.A., Raja F.A., Fotopoulou C., Gonzalez-Martin A., Colombo N., Sessa C., ESMO Guidelines Working Group (2018). Newly diagnosed and relapsed epithelial ovarian carcinoma: ESMO Clinical Practice Guidelines for diagnosis, treatment and follow-up. Ann. Oncol..

[23] Shang J., Han X., Zha H., Tao H., Li X., Yuan F., Chen G., Wang L., Ma J., Hu Y. (2021). Systemic Immune-Inflammation Index and Changes of Neutrophil-Lymphocyte Ratio as Prognostic Biomarkers for PATIENTSWITH Pancreatic Cancer Treated with Immune Checkpoint Blockade. Front. Oncol..

[24] Efil S.C., Guner G., Guven D.C., Celikten B., Celebiyev E., Taban H., Akyol A., Isik A., Kilickap S., Yalcin S. (2023). Prognostic and predictive value of tumor infiltrating lymphocytes in combination with systemic inflammatory markers in colon cancer. Clin. Res. Hepatol. Gastroenterol..

[25] Hudry D., Le Guellec S., Meignan S., Bécourt S., Pasquesoone C., El Hajj H., Martínez-Gómez C., Leblanc É., Narducci F., Ladoire S. (2022). Tumor-Infiltrating Lymphocytes (TILs) in Epithelial Ovarian Cancer: Heterogeneity, Prognostic Impact, and Relationship with Immune Checkpoints. Cancers.

[26] El Bairi K., Haynes H.R., Blackley E., Fineberg S., Shear J., Turner S., de Freitas J.R., Sur D., Amendola L.C., Masoumeh G. (2021). The tale of TILs in breast cancer: A report from the International Immuno-Oncology Biomarker Working Group. npj Breast Cancer.

[27] Miceska S., Skof E., Bucek S., Kuhar C.G., Gasljevic G., Smrkolj S., Prevodnik V.K. (2023). The prognostic significance of tumor-immune microenvironment in ascites of patients with high-grade serous carcinoma. Radiol. Oncol..

[28] Miceska S., Škof E., Gašljević G., Kloboves-Prevodnik V. (2023). Morphological and Immunocytochemical Characterization of Tumor Spheroids in Ascites from High-Grade Serous Carcinoma. Cells.

[29] Drakes M.L., Mehrotra S., Aldulescu M., Potkul R.K., Liu Y., Grisoli A., Joyce C., O’Brien T.E., Stack M.S., Stiff P.J. (2018). Stratification of ovarian tumor pathology by expression of programmed cell death-1 (PD-1) and PD-ligand- 1 (PD-L1) in ovarian cancer. J. Ovarian Res..

[30] Darb-Esfahani S., Kunze C.A., Kulbe H., Sehouli J., Wienert S., Lindner J., Budczies J., Bockmayr M., Dietel M., Denkert C. (2016). Prognostic impact of programmed cell death-1 (PD-1) and PD-ligand 1 (PD-L1) expression in cancer cells and tumor-infiltrating lymphocytes in ovarian high grade serous carcinoma. Oncotarget.

[31] Martin de la Fuente L., Westbom-Fremer S., Arildsen N.S., Hartman L., Malander S., Kannisto P., Måsbäck A., Hedenfalk I. (2020). PD-1/PD-L1 expression and tumor-infiltrating lymphocytes are prognostically favorable in advanced high-grade serous ovarian carcinoma. Virchows Arch. Int. J. Pathol..

[32] Thagaard J., Stovgaard E.S., Vognsen L.G., Hauberg S., Dahl A., Ebstrup T., Doré J., Vincentz R.E., Jepsen R.K., Roslind A. (2021). Automated Quantification of sTIL Density with H&E-Based Digital Image Analysis Has Prognostic Potential in Triple-Negative Breast Cancers. Cancers.

[33] Li J., Wang J., Chen R., Bai Y., Lu X. (2017). The prognostic value of tumor-infiltrating T lymphocytes in ovarian cancer. Oncotarget.

[34] Tubridy E.A., Eiva M.A., Liu F., Omran D.K., Gysler S., Brown E.G., Roy A.G., Zeng Y., Oh J., Cao Q. (2024). CD137+ tumor infiltrating lymphocytes predicts ovarian cancer survival. Gynecol. Oncol..

[35] Dai D., Liu L., Huang H., Chen S., Chen B., Cao J., Luo X., Wang F., Luo R., Liu J. (2021). Nomograms to Predict the Density of Tumor-Infiltrating Lymphocytes in Patients with High-Grade Serous Ovarian Cancer. Front. Oncol..

[36] Pizarro D., Romero I., Pérez-Mies B., Redondo A., Caniego-Casas T., Carretero-Barrio I., Cristóbal E., Gutiérrez-Pecharromán A., Santaballa A., D’Angelo E. (2023). The Prognostic Significance of Tumor-Infiltrating Lymphocytes, PD-L1, BRCA Mutation Status and Tumor Mutational Burden in Early-Stage High-Grade Serous Ovarian Carcinoma-A Study by the Spanish Group for Ovarian Cancer Research (GEICO). Int. J. Mol. Sci..

[37] Rathore A.S., Kumar S., Konwar R., Makker A., Negi M.P., Goel M.M. (2014). CD3^+^, CD4^+^ & CD8^+^ tumour-infiltrating lymphocytes (TILs) are predictors of favourable survival outcome in infiltrating ductal carcinoma of breast. Indian J. Med. Res..

[38] Gomez-Macias G.S., Molinar-Flores G., Lopez-Garcia C.A., Santuario-Facio S., Decanini-Arcaute H., Valero-Elizondo J., Treviño-Alvarado V., Ortiz-Lopez R., Dono A., Esteban-Zubero E. (2020). Immunotyping of tumor-infiltrating lymphocytes in triple-negative breast cancer and genetic characterization. Oncol. Lett..

[39] Aliyeva T., Aktas B.Y., Gundogdu F., Chalabiyev E., Arik Z., Usubutun A. (2024). The predictive role of PD-L1 expression and CD8^+^ TIL levels in determining the neoadjuvant chemotherapy response in advanced ovarian cancer. J. Ovarian Res..

[40] Karakaya A., Atıgan Y., Güler Ö.T., Demiray A.G., Bir F. (2021). The relation of CD3, CD4, CD8 and PD-1 expression with tumor type and prognosis in epithelial ovarian cancers. Ginekol. Pol..

[41] Buderath P., Mairinger F., Mairinger E., Böhm K., Mach P., Schmid K.W., Kimmig R., Kasimir-Bauer S., Bankfalvi A., Westerwick D. (2019). Prognostic significance of PD-1 and PD-L1 positive tumor-infiltrating immune cells in ovarian carcinoma. Inter. J. Gynecol. Cancer.

[42] Baş Y., Koç N., Helvacı K., Koçak C., Akdeniz R., Şahin H.H.K. (2021). Clinical and pathological significance of programmed cell death 1 (PD-1)/programmed cell death ligand 1 (PD-L1) expression in high grade serous ovarian cancer. Transl. Oncol..

[43] Sawada M., Goto K., Morimoto-Okazawa A., Haruna M., Yamamoto K., Yamamoto Y., Nakagawa S., Hiramatsu K., Matsuzaki S., Kobayashi E. (2020). PD-1+ Tim3+ tumor-infiltrating CD8 T cells sustain the potential for IFN-γ production, but lose cytotoxic activity in ovarian cancer. Int. Immunol..

[44] Koh C.H., Lee S., Kwak M., Kim B.S., Chung Y. (2023). CD8 T-cell subsets: Heterogeneity, functions, and therapeutic potential. Exp. Mol. Med..

[45] Pinto M.P., Balmaceda C., Bravo M.L., Kato S., Villarroel A., Owen G.I., Roa J.C., Cuello M.A., Ibañez C. (2018). Patient inflammatory status and CD4^+^/CD8^+^ intraepithelial tumor lymphocyte infiltration are predictors of outcomes in high-grade serous ovarian cancer. Gynecol. Oncol..

[46] Barna A.J., Herold Z., Acs M., Bazsa S., Gajdacsi J., Garay T.M., Herold M., Madaras L., Muhl D., Nagy A. (2023). High Tumor-Infiltrating Lymphocyte Count Is Associated with Distinct Gene Expression Profile and Longer Patient Survival in Advanced Ovarian Cancer. Int. J. Mol. Sci..

[47] Preston C.C., Maurer M.J., Oberg A.L., Visscher D.W., Kalli K.R., Hartmann L.C., Goode E.L., Knutson K.L. (2013). The ratios of CD8^+^ T cells to CD4^+^CD25+ FOXP3+ and FOXP3- T cells correlate with poor clinical outcome in human serous ovarian cancer. PLoS ONE.

[48] Farolfi A., Scarpi E., Greco F., Bergamini A., Longo L., Pignata S., Casanova C., Cormio G., Bologna A., Orditura M. (2020). Inflammatory indexes as predictive factors for platinum sensitivity and as prognostic factors in recurrent epithelial ovarian cancer patients: A MITO24 retrospective study. Sci. Rep..

[49] Mao H., Yang F. (2023). Prognostic significance of systemic immune-inflammation index in patients with ovarian cancer: A meta-analysis. Front. Oncol..

[50] Chu B., Chen Y., Pan J. (2025). Prognostic significance of systemic immune inflammation index for ovarian cancer: An updated systematic review and meta-analysis. J. Ovarian Res..

[51] Nie D., Gong H., Mao X., Li Z. (2019). Systemic immune-inflammation index predicts prognosis in patients with epithelial ovarian cancer: A retrospective study. Gynecol. Oncol..

[52] Kuang T., Qiu Z., Wang K., Zhang L., Dong K., Wang W. (2024). Pan-immune inflammation value as a prognostic biomarker for cancer patients treated with immune checkpoint inhibitors. Front. Immunol..

[53] Chi D.S., Venkatraman E.S., Masson V., Hoskins W.J. (2000). The ability of preoperative serum CA-125 to predict optimal primary tumor cytoreduction in stage III epithelial ovarian carcinoma. Gynecol. Oncol..

[54] Bachmann R., Brucker S., Stäbler A., Krämer B., Ladurner R., Königsrainer A., Wallwiener D., Bachmann C. (2021). Erratum: Prognostic relevance of high pretreatment CA125 levels in primary serous ovarian cancer. Mol. Clin. Oncol..

[55] Ay S., Ozyukseler D.T., Dulgar O., Basak M., Yildirim M.E., Gumus M. (2021). Initial CA125 value as a predictive marker for high-grade serous ovarian cancer. J. Coll. Physicians Surg. Pak..

[56] Asali A., Haj-Yehia N., Zehavi T., Perry T., Beiner M., Fishman A., Kadan Y. (2021). High grade, advanced, serous ovarian cancer with low serum CA125 levels. J. Obstet. Gynaecol..

[57] Ikeda A., Yamaguchi K., Yamakage H., Abiko K., Satoh-Asahara N., Takakura K., Konishi I. (2020). Serum lactate dehydrogenase is a possible predictor of platinum resistance in ovarian cancer. Obstet. Gynecol. Sci..

[58] Bastani A., Asghary A., Heidari M.H., Karimi-Busheri F. (2017). Evaluation of the sensitivity and specificity of serum level of prostasin, CA125, LDH, AFP, and hCG+β in epithelial ovarian cancer patients. Eur. J. Gynaecol. Oncol..

[59] Yang D., Li H., Sun X., Yang S., Wang K., Liu Z. (2020). Clinical usefulness of high levels of C-reactive protein for diagnosing epithelial ovarian cancer. Sci. Rep..

