# Supplementary material for: Association of Tumor-Infiltrating Lymphocytes and Inflammation Status with Survival Outcome in Patients with High-Grade Serous Ovarian Carcinoma
Source: Cancers (Basel). 2025 Jul 8;17(14):2269. doi: 10.3390/cancers17142269 (PMC12293900; doi:10.3390/cancers17142269)
Supplement: Supplementary file 1 [file cancers-17-02269-s001.zip › cancers-3694148-supplementary.pdf]

cancers

MDPI

Supplementary Materials: Association of Tumor-Infiltrating Lymphocytes and Inflammation Status with Survival Outcome in Patients with High-Grade Serous Ovarian Carcinoma

Simona Miceska, Cvetka Grašič Kuhar, Snježana Frković Grazio, Erik Škof, Praveen Krishnamoorthy, Dineo Khabele and Veronika Kloboves Prevodnik

Table S1. Individual score of the manual assessment of TILs of both investigators (S.F.G. and S.M.).

	TILs subtype	Investigatior 1 (S.F.G.) median % (range min-max)	Investigator 2 (S.M.) median % (min-max)
**iTILs**	CD3+ iTILs	1.83 (0.00–20.00)	1.00 (0.00–20.00)
	CD4+ iTILs	0.33 (0.00–6.67)	0.33 (0.00–8.33)
	CD8 iTILs	1.00 (0.00–20.00)	1.00 (0.00–16.67)
	PD-1 iTILs	0.02 (0.00–5.00)	0.01 (0.00-5.00)
**sTILs**	CD3+ sTILs	13.33 (0.33–70.00)	15.00 (0.33–76.67)
	CD4+ sTILs	3.67 (0.17–46.67)	2.33 (0.17–46.67)
	CD8+ sTILs	8.67 (0.17–43.33)	8.67 (0.17–43.33)
	PD-1 + sTILs	0.00 (0.00–2.00)	0.0 (0.00.2.009

Table S2. Association between CD8+ sTIL and the other clinical characteristics/inflammation status.

Variable	Chi-square	Asymptotic p-value	2-sided exact p-value	1-sided exact p-value
**FIGO Stage**	1.103	0.294	0.410	0.283
**Residual disease**	3.720	0.054	0.083	0.058
**SII**	0.038	0.845	1.000	0.586
**PIV**	0.430	0.512	0.665	0.399
**CA 125**	2.880	0.090	0.135	0.095
**LDH**	0.135	0.714	1.000	0.497
**CRP**	4.800	0.028	0.065	0.032

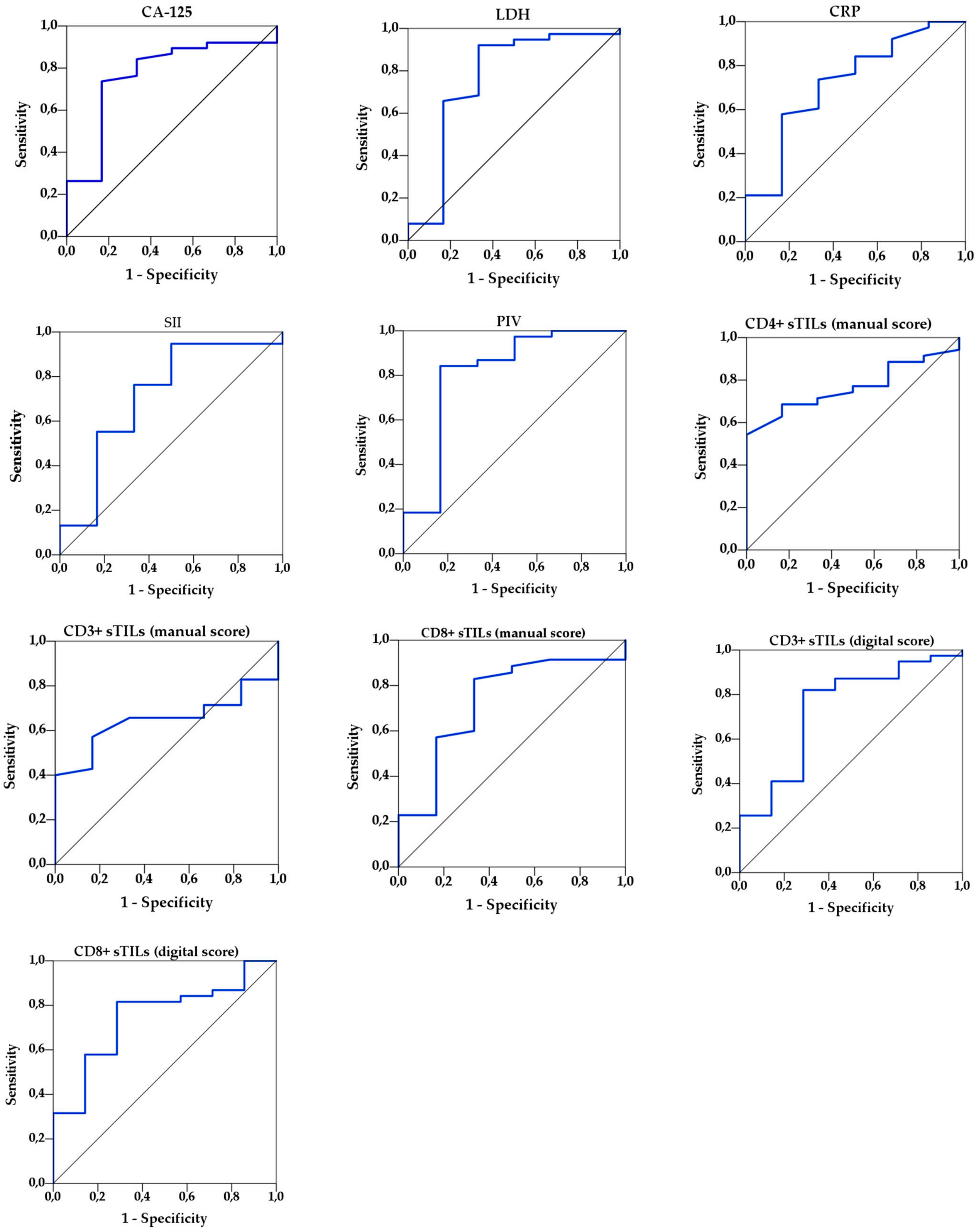

Figure S1. ROC curves for the analyzed parameters to determine the cut-off values for classifying low vs. high groups.
